# 2684. Characteristics of mpox patients treated with Tecovirimat: a single center review

**DOI:** 10.1093/ofid/ofad500.2295

**Published:** 2023-11-27

**Authors:** Robert Vargas, Jennifer Fernandez, David Taylor, Tondria Green, Sarah Y Won, Beverly E Sha, Laura Hernandez Guarin, Shivanjali Shankaran

**Affiliations:** Rush University Medical Center, Chicago, Illinois; Rush University Medical Center, Chicago, Illinois; Rush Medical College, Chicago, Illinois; Rush University Medical Center, Chicago, Illinois; Rush University Medical Center, Chicago, Illinois; Division of Infectious Diseases, Department of Internal Medicine, Rush University Medical Center, Chicago, IL, USA, Chicago, Illinois; Rush University Medical Center, Chicago, Illinois; Rush University Medical Center, Chicago, Illinois

## Abstract

**Background:**

The 2022 mpox outbreak disproportionately affected men who have sex with men (MSM), and communities of color. Tecovirimat, which was FDA-approved for the treatment of smallpox in 2018, was made available to treat mpox via an Expanded Access Investigational New Drug protocol through the Centers for Disease Control (CDC). This retrospective chart review describes the demographics and characteristics of patients treated with tecovirimat at a single academic hospital in Chicago, IL.

**Methods:**

The charts of patients with suspected or confirmed mpox infection who received tecovirimat were retrospectively reviewed. Demographics, HIV status, mpox vaccination, presentation and outcome were collected.

**Results:**

Tecovirimat was prescribed to 22 patients. The average patient age was 38 years (26-55 years), 73% were nonwhite: 10 identified as Black and 6 as Hispanic. 91% were MSM, and 9% identified as heterosexual. 55% (12/22) had a known diagnosis of HIV: 42% (5/12) of this cohort were not on antiretroviral therapy, and 3 had a CD4 count < 200. 73% of all patients had at least 1 additional sexually transmitted infection (STI), the most common were syphilis (7/22), gonorrhea (3/22) and/or chlamydia (3/22). 16 patients had not received any doses of the vaccine. 64% had > 10 lesions, and 68% had systemic symptoms of headache, fevers or myalgias. 10 patients were treated as outpatients, 12 needed hospital admission and one needed an ICU stay for IV tecovirimat. Follow-up was available for 16 patients; all but two completed their treatment. Only one person stopped tecovirimat due to mild gastrointestinal side effects. Other than one patient who took > 6 months to recover, symptoms had completely resolved at a median of 18 days (8-30 days) from positive test result.

**Figure:**
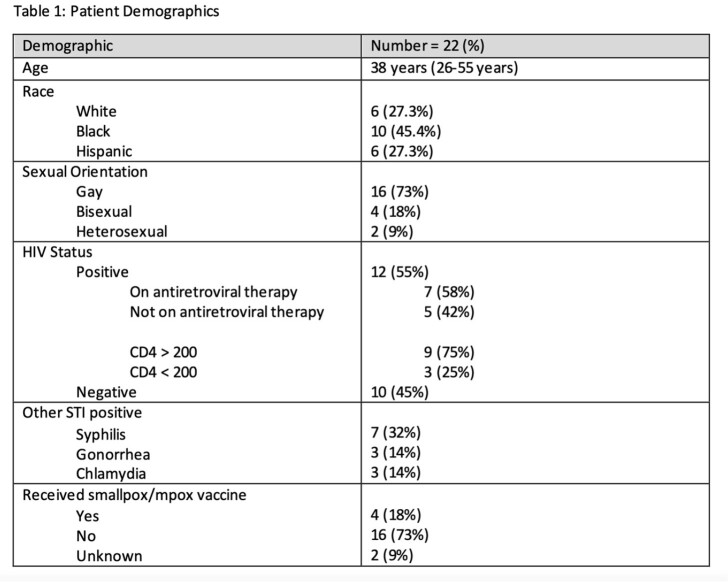

**Figure:**
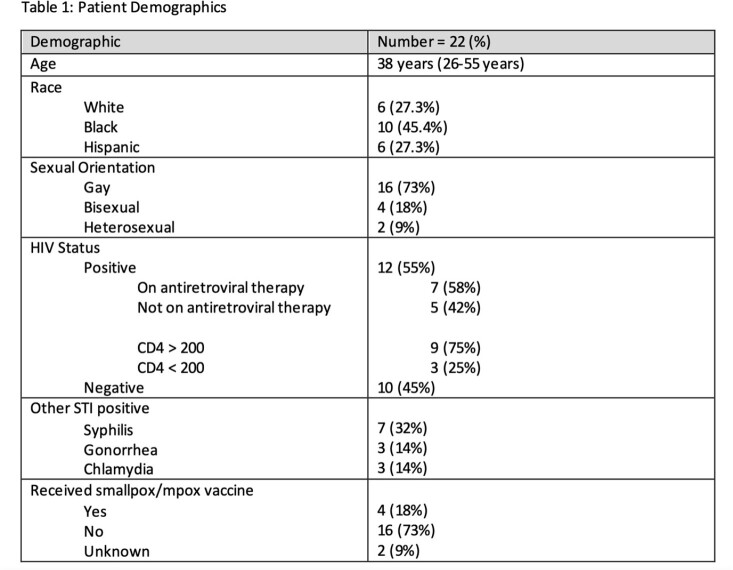

**Conclusion:**

At our institution, patients receiving tecovirimat were predominantly MSM and nonwhite. A majority had other STIs and/or had a diagnosis of HIV, emphasizing the importance of STI testing, and providing a potential opportunity to reengage people in HIV care. The retrospective nature of our study limits assessment for treatment efficacy. However, tecovirimat was well tolerated, with only one person having mild side effects. This suggests that tecovirimat may play an important role in the management of patients with mpox infection.

**Image 1.**
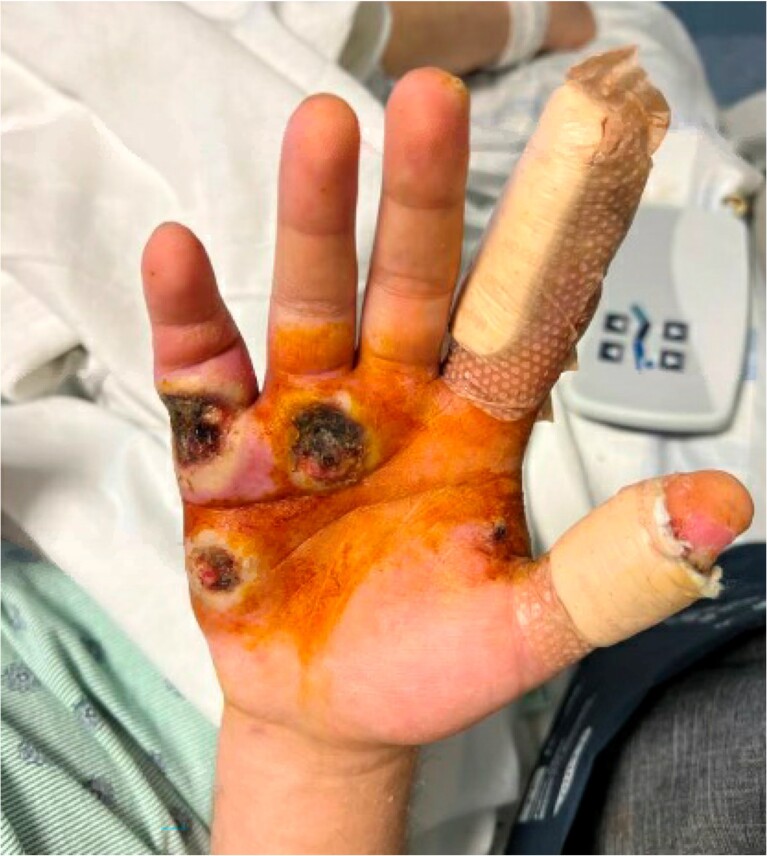
Example of mpox lesions

**Image 2.**
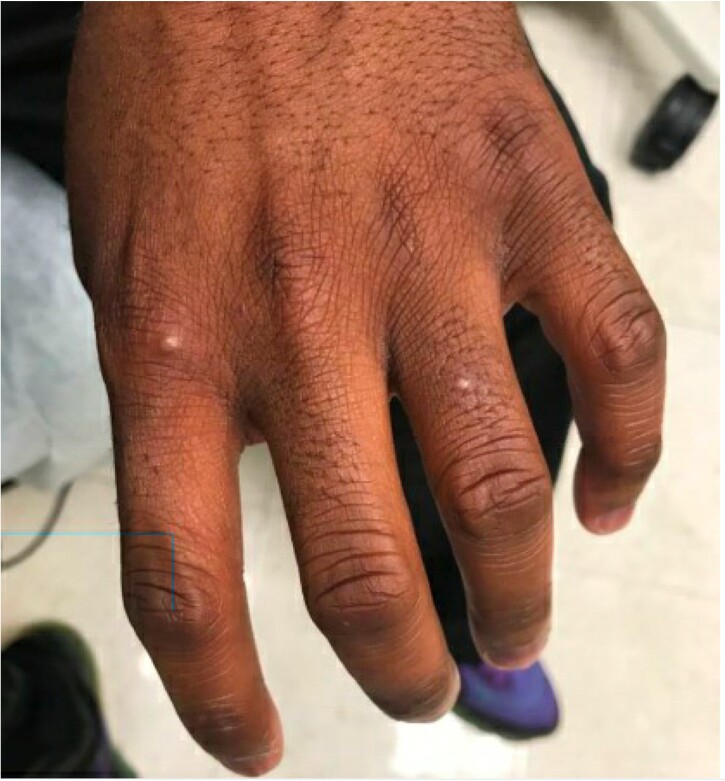
Example of mpox lesions

**Disclosures:**

**All Authors**: No reported disclosures

